# Health insurance literacy and health services access barriers in Niemann–Pick disease: the patient and caregiver voice

**DOI:** 10.1186/s13023-022-02490-8

**Published:** 2022-09-02

**Authors:** George A. Diaz, Joslyn Crowe, Justin Hopkin

**Affiliations:** 1 Division of Medical Genetics and Genomics, Department of Genetics and Genomic Sciences, Icahn School of Medicine at Mount Sinai, 1428 Madison Avenue, 1st Floor, Room AB1-12, New York, NY 10029 USA; 2 National Niemann-Pick Disease Foundation, Fort Atkinson, WI USA

**Keywords:** Niemann–Pick disease, Health insurance literacy, Access challenges

## Abstract

**Background:**

Major challenges to health care access include low health insurance literacy, prohibitive costs, and insurance barriers. Niemann–Pick disease (NPD), comprising acid sphingomyelinase deficiency (ASMD) and Niemann–Pick type C (NPC), is a group of rare, autosomal recessive, highly heterogeneous, neurovisceral, life-threatening, relentlessly progressive lysosomal disorders. Patients experience debilitating systemic and neurological symptoms and substantial emotional and financial stress. Currently, these multifaceted disorders are managed symptomatically as there are no approved therapies. Given the considerable disease burden of NPD, timely access to quality health care is paramount for improving outcomes in these life-threatening disorders. Understanding health insurance literacy and access challenges among patients with NPD and their caregivers is a first step to overcoming treatment barriers.

**Results:**

Patients from the Niemann–Pick community participated in a health insurance literacy survey and follow-up telephone interviews on perceived access challenges. Of the 79 respondents who completed the survey, 67 participated in interviews. All respondents had stable health insurance coverage. However, 61% of respondents were unaware of Medicaid waivers and did not avail of them. Overall, 50% of respondents with childhood onset NPC selected Medicaid/Medicare and private insurance; 35% utilized Medicaid waivers. Most respondents with ASMD had private insurance only. Although the Niemann–Pick community demonstrated greater health insurance literacy than the general population, knowledge gaps exist in calculating insurance coverage, out-of-pocket maximums, and defining a formulary. The most frequently cited access burden was the process of obtaining medical care and services. Among respondents with ASMD, the greatest access burden was fear of unavailability of or access to medications and treatment. Access challenges adversely impacted patients’ mental health and exacerbated physical symptoms. Delays and denials in obtaining essential medication, equipment, and services contributed to disease progression. Caregivers faced burnout and often questioned the utility of their advocacy.

**Conclusions:**

This study identified knowledge gaps in health insurance literacy and challenges to access medication and health care services among individuals impacted by NPD. Patients and caregivers need the knowledge and skills to navigate a complicated health care system, understand their rights to medication and services and, ultimately, benefit from improved outcomes, especially in a post–drug approval era.

## Background

Health insurance literacy is defined as “the degree to which individuals have the knowledge, ability, and confidence to find and evaluate information about health plans, select the best plan for their own (or their family's) financial and health circumstances, and use the plan once enrolled” [1]. The US has one of the most complex systems in the world for paying for health care [1]. A myriad of health insurance plans are available in the US health care system, and selecting a suitable health insurance plan is a major health and financial decision [2]. Plans that provide optimal coverage at low cost (cost-effective) can be beneficial to insured individuals. Most adults purchase their own insurance or utilize employer-provided health insurance for themselves or their dependents. However, the processes of selecting and enrolling in an insurance plan are complicated and can be daunting because of the use of unfamiliar insurance terms, complex provisions, and rules [1, 2]. Inadequate health insurance literacy adversely affects ease of selecting health insurance plans and providers, obtaining enrollment, and awareness of reforms and increases financial hardship [1, 3]. Therefore, an urgent unmet need exists for educating individuals in health insurance concepts. Furthermore, most individuals are restricted to insurance plans offered by their employer, and they may not have the option of choosing a program that will accommodate their unique requirements. Therefore, individuals must be equipped with the skills to understand and navigate insurance plans that do not fulfill their medical needs so that they can better utilize available health care benefits and services.

Health insurance literacy also needs to translate into effective health care utilization so that maximum benefits can be accessed. A systematic review showed that greater health insurance literacy was associated with more efficient and cost-effective health care utilization [4]. Insufficient health insurance literacy can also negatively impact health-related quality of life and contribute significantly to an individual’s health status [5–8].

Health insurance barriers are amplified in rare diseases in which access to the very few experts, affiliated with a small number of centers of excellence, may not be covered by the selected insurance plan [9]. Less than 5% of rare diseases have treatments available [10]. Treatments often are specialty drugs that are expensive [9]. For many rare diseases, there are no disease management guidelines making it difficult for caregivers to demonstrate the need for supportive services and insurance coverage [9]. One study estimated medical care costs for rare diseases as 3- to 5-fold greater than those for age-matched controls without a rare disease [11]. Often, a primary insurance plan is insufficient to cover expenses, and families supplement their coverage with secondary insurance or other financial assistance options [9]. Parents of children with rare diseases have also expressed the challenges associated with completing confusing insurance benefits documents, obtaining secondary insurance, and having to repeatedly interact with insurance representatives while simultaneously caring for their children [9].

Niemann–Pick disease (NPD) is a group of rare, autosomal recessive, highly heterogeneous, neurovisceral, progressive lysosomal disorders that are often life-threatening [12, 13]. NPD types A and B, also known as acid sphingomyelinase deficiency (ASMD), manifest as a continuum of phenotypes with varying severity and occur due to the abnormal accumulation of sphingomyelin in different organs such as the spleen, liver, lung, and bone marrow [13–15]. In severe forms, the nervous system is also involved [13, 14]. Infantile neurovisceral (acute) ASMD (type A) is the most severe, rapidly progressive form, and patients do not survive beyond 2 to 3 years [13, 15–17]. Chronic neurovisceral (intermediate form, type A/B) and chronic visceral (type B) ASMD are slowly progressive forms with symptom onset occurring from childhood through adulthood [14]. In these forms, death occurs most frequently due to complications such as liver or respiratory failure, especially in the pediatric population (< 21 years of age) [18, 19].

NPD type C (NPC) occurs due to impaired intracellular trafficking and abnormal lysosomal accumulation of unesterified cholesterol and other lipids in the liver, spleen, brain, and other tissues [12]. Disease severity and progression depend on neurological involvement [20]. NPC has a highly heterogeneous clinical presentation. Phenotypes range from a rapidly progressive, fatal, neonatal disease to a late infantile form with gait problems and language delays, a juvenile or classic form characterized by ataxia and significant executive function and cognitive impairment, and an adult onset form in which cognitive impairment and coexisting psychiatric illness followed by neurological symptoms are more frequently reported [12, 20–22]. Regardless of age at onset of neurological symptoms, all forms are neurologically progressive and fatal [12, 21]. Most patients die between 10 and 25 years of age; however, the early infantile form causes death by 5 years [12, 20].

NPD is associated with substantial disease burden [21, 23]. Patients and caregivers undergo considerable long-term physical, emotional, psychosocial, and financial distress [21, 24, 25]. Caregivers of patients with ASMD experienced significant financial burden and insecurity attributed to high out-of-pocket medical expenses, lack of insurance coverage for supportive care, emergency hospitalizations, reduced working hours, or the necessity of working at an undesired job due to the fear of losing health insurance [21, 24]. NPC impacts activities of daily living (ADLs) such as eating and drinking, self-care, ambulation, communication, and participation in school and work. Caregivers of patients with NPC faced additional demands on their time and their physical and mental strength and felt constant anxiety and heightened concern [21]. Available data available show that the financial burden is considerable among patients with NPC and their families [26]. Residential care, home services, disease progression, and patient or caregiver employment loss or decreased working hours can substantially inflate total costs and reduce income among families impacted by NPC [26].

Currently, there are no US Food and Drug Administration (FDA)–approved disease-modifying therapies for ASMD and NPC [20, 22, 25]. To minimize the impact of symptoms and prevent disease complications, treatment of these multisystem conditions currently involves symptom-specific treatment and supportive or palliative care [20, 22, 25]. Supportive care is provided by a multidisciplinary team of physicians, including a hepatologist or gastroenterologist, neurologist, ophthalmologist, pulmonary or critical care physician, endocrinologist, physical therapist, and geneticist [27, 28]. A lead consultant coordinates patient care, while a nurse specialist and social worker educate and assist patients and their caregivers and provide adequate support and financial resources to complete treatment [27]. Olipudase alfa enzyme replacement therapy for ASMD and miglustat are approved by the European Medicines Agency and in other countries [20, 29]. These drugs and other emerging treatments such as 2-hydroxypropyl-beta-cyclodextrin, leucine-IB 1001, and arimoclomol for NPC have been granted orphan drug status by the FDA [13, 20, 21]. In the US, miglustat is currently used off-label for NPC [20].

Given the complex health care system and the devastating clinical course and chronic progressive nature of ASMD and NPC, health care access barriers—especially health insurance barriers—impose an additional burden on patients and caregivers. The anticipated approval of new therapies for ASMD and NPC makes it imperative to sufficiently equip patients and their caregivers with the knowledge and tools to navigate a complicated insurance system of cumbersome processes and paperwork and facilitate access to care, medication, and health services, especially in a post-approval era, where medication access may be restricted by prohibitive costs and complicated insurance systems [26]. In this context, it is of utmost importance to understand the unmet needs of patients and caregivers as a first step [21].

This paper presents the findings of a US-based qualitative patient-reported outcomes (PRO) study to assess health insurance literacy and perceived challenges in accessing health care, services, and treatment/medications among patients with NPD and their families/caregivers (NCT04469894). The results of this study are expected to identify gaps in health care access among patients with NPD and better inform initiatives, programs, and resources designed to assist patients living with NPD and their families.

## Results

### Patient demographics

A total of 79 out of 410 individuals (19.3%) of the US Niemann–Pick community invited to participate in the study completed the quantitative survey. Of these, 67 (84.8%) participated in the structured interviews. A large majority of surveys were completed by caregivers (parents/guardians) who responded on behalf of children or adults who were unable to directly provide answers to the survey or interview questions.

**Fig. 1:**
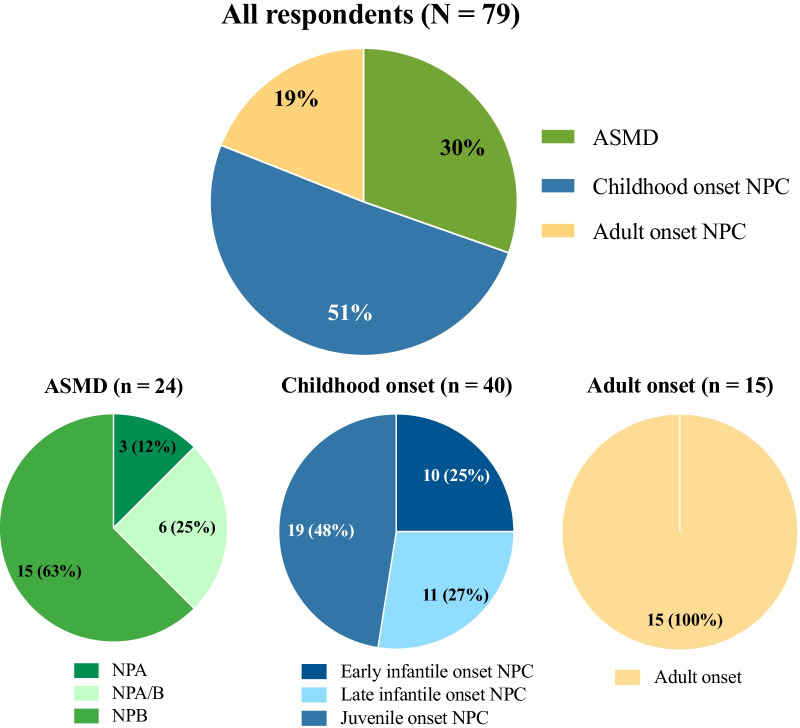
Patient demographics by disease subtype (N = 79)

NPC is generally categorized into perinatal, early infantile (2 months to 2 years), late infantile (2–6 years), juvenile or classic (6–15 years), and adolescent and adult forms (> 15 years) [12]. For this analysis, patients with early infantile, late infantile, or juvenile forms of NPC were grouped under “childhood onset NPC.” Overall, 51% of the patients had childhood onset NPC, 19% had adult onset NPC, and 30% had ASMD. The chronic visceral form of ASMD (63%) and juvenile form of NPC (48%) accounted for most of the cases of ASMD and NPC, respectively (Fig. 1). The distribution of patients was representative of the general spectrum of NPD subtypes and clinical forms and geographic distribution.

The majority of patients (74%) were diagnosed within the last 9 years and 34% within the last 3 years. Consistent with the rapid clinical course of the disease, patients with acute neurovisceral ASMD died at a younger age (an average of 2.25 years) than those with childhood onset NPC (an average of 13.56 years). All adults with NPC who participated in the study were living at the time of the study.

### Health insurance status and types

**Fig. 2:**
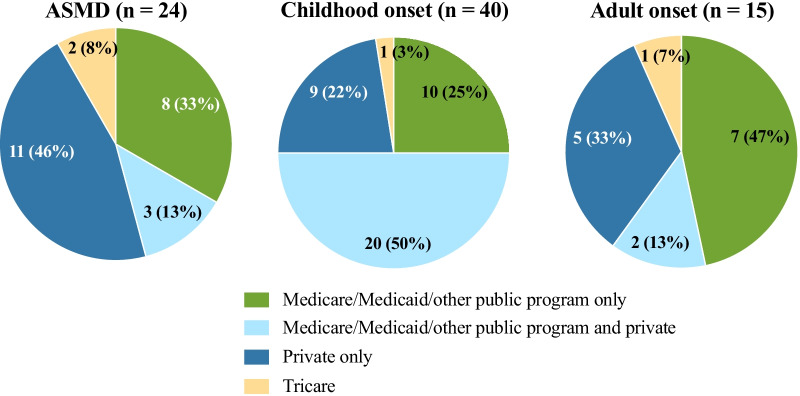
Insurance types by disease group (N = 79)

The online survey showed that respondents utilized several different health insurance programs and in a variety of combinations (Fig. 2). Although respondents were cognizant of the names of their insurance programs, they were unable to categorize them by type as state, private, or Tricare. Half the patients with childhood onset NPC enrolled in a combination of Medicare/Medicaid/other public programs and private insurance, whereas most patients with ASMD (46%) selected private insurance only. Patients with adult onset NPC were most likely to use state-provided insurance (47%).

### Health insurance literacy

Respondents had a median score of 75% (range 17–100%) on the health insurance literacy quiz and 78% (range 10–100%) on the self-assessment ratings. In general, quiz scores were consistent with respondents’ average self-assessment rating. Higher quiz scores were positively correlated with higher self-rating scores, and this result was statistically significant (*P* < 0.005). Respondents who rated themselves as high in any particular skill domain (information-seeking skills, document literacy skills, and cognitive skills) scored high in other skill domains as well.

**Fig. 3:**
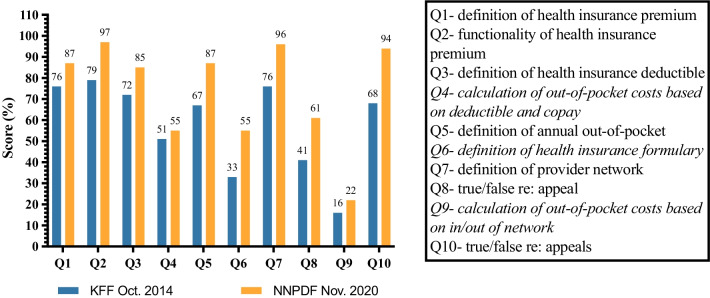
Responses to the health insurance terminology quiz (n = 68)

On average, respondents from the NPD community scored higher on the health insurance literacy quiz than the population in the Kaiser Family Foundation study, which was representative of the general US population (Fig. 3). However, knowledge gaps were apparent among respondents in calculating out-of-pocket costs based on co-pays and deductibles and in- or out-of-network insurance. Furthermore, 55% of the respondents were unable to define a health insurance formulary. A formulary (or a preferred drug list) is a continually updated list of generic or brand-name drugs that are approved by a specific health insurance provider [30].

### Health insurance coverage attributes

All respondents had stable health insurance coverage; most did not change their provider over the year. Among respondents with private insurance, 48 of 51 (94%) obtained insurance via their employer. Of 46 respondents for whom employers partially paid the premium, 26 did not know the amount paid by their employers.

**Fig. 4:**
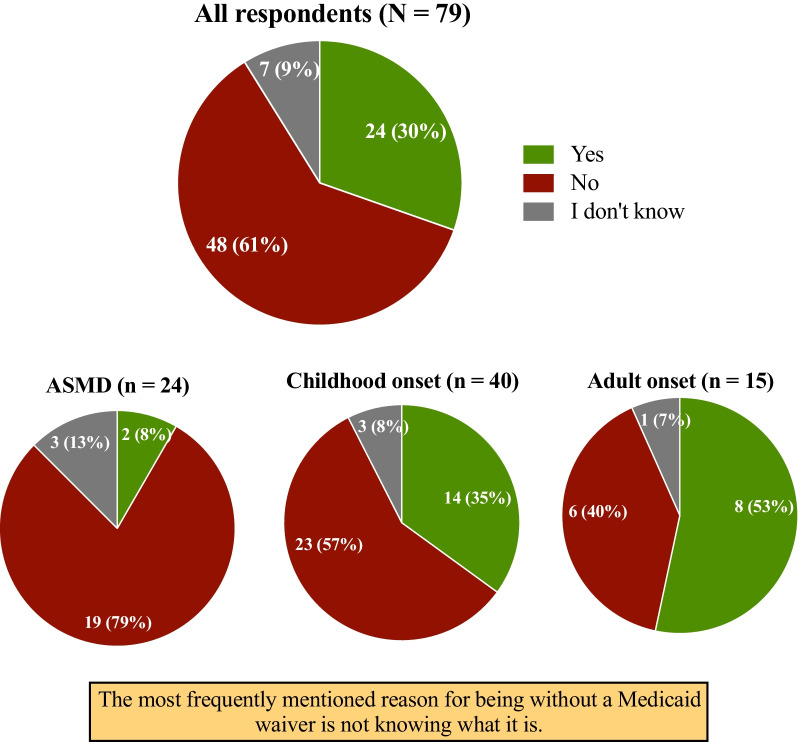
Patients utilizing Medicaid waivers (N = 79)

Most respondents (90%) reported low difficulty in covering medical expenses. However, more than half (48/79, 61%) of the respondents did not have Medicaid waivers; the most frequent reason was lack of awareness followed by a lack of clarity on the requirements to qualify for a waiver (Fig. 4). Medicaid waivers pay for standard medical and nonmedical individualized health care of older adults and individuals with disabilities or chronic health conditions for inpatient care in their home or community [31]. Individuals with a demonstrated functional and financial need can apply for home- and community-based service waivers (e.g., case management, home health aides, respite care, home/vehicle modifications, durable medical equipment) [31]. More patients with adult onset NPC (53%) and childhood onset NPC (35%) availed of a Medicaid waiver than did patients with ASMD (8%). Respondents who availed of Medicaid waivers did so to cover home health care (12 mentions) and hours of respite care (10 mentions) but did not use all services offered.

Most respondents reported their mean individual and family deductibles and out-of-pocket maximums as < $2000 and < $3000, respectively. Almost half (47%) of the respondents did not perceive their deductibles as high and met their individual deductible within the first quarter of the year. However, inconsistent responses to questions on deductibles, out-of-pocket costs, and out-of-pocket-maximums, including some responses indicating that respondents had no deductibles or out-of-pocket maximums, revealed knowledge gaps and a lack of understanding of these concepts. In general, preferred brand-name drugs had the highest co-pay amount of up to $2000, whereas generic drugs had the lowest co-pay amount of a maximum of $10. The co-pay cost for miglustat, which is used off-label to treat NPC, was as much as $1800 per month for some patients.

Most respondents did not use a health savings account (68%) or seek financial assistance from co-pay support programs (92%), reduced payment programs (97%), free drug programs (95%), or nonprofit organizations (94%).

### Access challenges

**Fig. 5:**
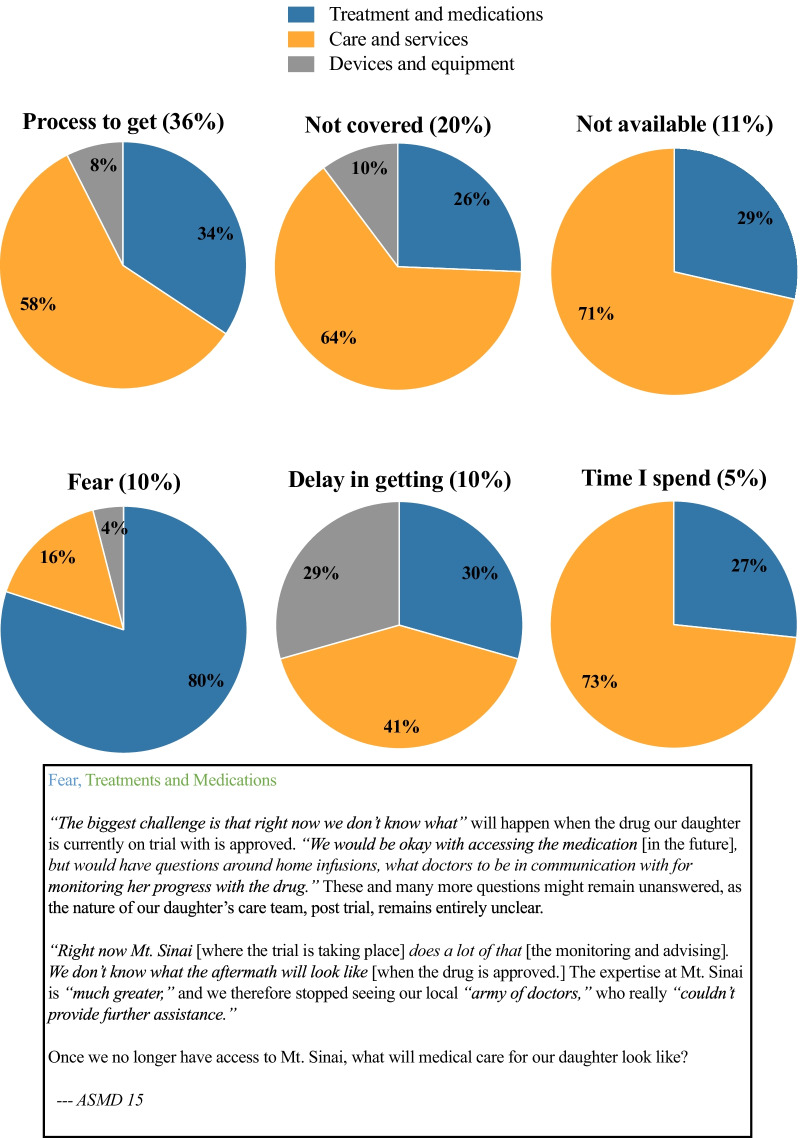
Challenges to access health care services (n = 67)

In the qualitative interview, in general and across all NPD subtypes, respondents perceived the process to obtain medical care, treatment, and services as the greatest access challenge (36% of responses) (Fig. 5). As a parent of a patient with juvenile onset NPC explained, “*There was a lot—had to fight so hard to get her anything; we had big time trouble getting a waiver to get nursing in house. Found out we didn’t have to fight it—they weren’t supposed to deny us; we are one of the 30 rarest diseases. When I found out, it was after the fact—she wasn’t bed bound yet, didn’t have certain machines, she had a g-tube, didn’t understand why we couldn’t get help because they typically provide this—we finally did, after that, everything was covered but it was a hard fight*.”

Other frequently reported access challenges included health care and services not being covered (20%) or being unavailable (11%) as well as fear of treatment and medications being unavailable or not being covered by insurance (10%) (Fig. 5). The fact that this fear was grounded in harsh realities is exemplified by a parent of a patient with adult onset NPC: “Coverage of physical therapy and occupational therapy—the OT [occupational therapist] felt [my son] should continue. However, [the OT’s] services … were not covered … because Medicare felt that [my son] had maxed out. We feel that therapy should continue, but once therapies are ‘maxed out,’ you have to prove progress, or they will not be covered. He still needs something; one facility has a rehab gym. You can pay a small fee for the gym, there is an exercise physiologist who runs it, but that [fee] was on us. If it was just [our son] doing it [on his own], [the exercise] would have never happened. [Our son] is in OT and PT again; this is a new period; we assume it will last through August and we will reassess again.” A genuine concern across NPD subtypes was the uncertainty surrounding access to and availability of medication, services, and experts in the field after the end of the clinical trial in which their child was enrolled (Fig. 5).

The second most-commonly perceived access burden varied across NPD subtypes. Respondents affected by ASMD reported higher rates (19%) of fear of being unable to access treatment or services than respondents affected by NPC (10% for childhood onset NPC and 7% for adult onset NPC). Respondents affected by NPC most frequently rated care and services not being covered by insurance as their second-highest access burdens (20% for childhood onset NPC and 23% for adult onset NPC). In general, respondents with state and private insurance had to struggle to obtain what they required; with state insurance, the struggle was less severe but there were greater delays.

### Impact of access challenges on daily living

Patients and caregivers reported being consistently adversely impacted by the challenges in accessing health care and feared delays in obtaining medical care and services. Access challenges primarily affected patients’ mental health (39 mentions) and physical symptoms (25 mentions). Patients most frequently experienced increased stress, fear, and depression. The most-commonly affected physical symptoms were issues with balance; difficulties with movement, walking, and ADLs; fatigue; and cognitive impact. Furthermore, delays and denials of medication, equipment, or services led to discomfort, disease progression, and increased expenditure (Table 1). The gravity of these obstacles is underscored in a statement by a parent of a child with infantile onset NPC: “*We did not get the treatment during Covid—March, April, May. During that span—she just turned 9 yesterday [and] has been getting it consistently since she was 4—we noticed a major decline in walking, talking, her cognitive, also started having seizures—tonic–clonic, very bad seizures, the worst thing I've ever seen.*”

**Table 1:** Patient-reported life impacts from access burdens

Life impact	Number of mentions
Mental health	39
Physical symptoms	25
Impact from delays and denials	16
Future impact	13
Caregiver: support system	10
Independence	8
Social impacts	8
Access challenges regarding quality of care	8
Financial	5
Traveling difficulties	5
Misses school/events	4
Caregiver mental health	3

Access challenges also had a substantial negative impact on caregivers/parents. Caregivers felt overwhelmed and faced burnout (14 mentions) as they were required to be constantly vigilant and provide 24-h care in addition to caring for other siblings and managing work. A parent of a child with juvenile onset NPC captured the commitment and love that accompany living with these rare diseases. “*The challenge is great but my love and strength for my son are greater. I will never stop fighting to get any treatment that could help my son and this terrible, unknown disease. I plan on spending the rest of my life taking care of him. Right now, I’m on call 24/7, 365 days of the year.*” Caregivers also had serious concerns about whether they were advocating in the right way (14 mentions); this included making informed decisions, staying updated with new research and information, knowing what is covered, and being aware of available yet limited resources (Table 2).

**Table 2:** Caregiver-reported life impacts from access burdens

Life impact	Number of mentions
Caregiver burnout	14
Advocating in the right ways	14
Emotional toll	9
Filing appeals	8
Providing financial support	7
Accessing treatments	3
Awareness of resources	3
Learning to care for child who is losing abilities	2
Power of attorney confusion	2

## Discussion

To the best of our knowledge, this is the first study assessing health insurance literacy and access challenges among patients with NPD.

About 20% of the members of the US Niemann–Pick community who were invited to participate in the study completed the online survey. This response rate, although relatively low, is expected for surveys of this type. More importantly, the survey participants represented the current Niemann–Pick community in the US that is actively managing and living with the disease and its complexities. Caregivers responded to the survey and interview questions on behalf of children or adults who were unable to directly provide answers themselves. Although actual numbers are unavailable, majority of surveys were completed by caregivers, and the number of patients providing direct input was small and is expected to be biased toward patients with late adult onset NPC and late ASMD.

The distribution of survey respondents was representative of NPD subtypes, with the chronic visceral form of ASMD and juvenile form of NPC accounting for most of the cases [12, 14]. As one-third of the patients were diagnosed within the last 3 years, and most patients within the last 10 years, the patient experience was contemporary. The age at death of deceased patients in each NPD subtype reflected disease progression. Patients with acute neurovisceral ASMD died at a younger age (an average of 2.25 years). These results are consistent with previously published results for this form of ASMD and reflect its devastating, rapidly deteriorating, and fatal clinical progression [17].

The online health insurance literacy quiz and self-assessment indicated that the NPD community had greater health insurance literacy and was more knowledgeable than the general population. These results may be expected of individuals living with a chronic disease and the necessity of navigating the health care system. In addition, this may reflect a selection bias of survey respondents comprising mainly members of the National Niemann–Pick Disease Foundation (NNPDF) and other NPD organizations, who may have greater access to pertinent information and are therefore relatively well informed. However, significant knowledge gaps were observed in calculating out-of-pocket costs and maximums and defining formularies. Currently, no treatments have been approved by the FDA for NPD, and therefore, they are not yet on the formulary, which may contribute to respondents’ lack of familiarity with this term. These findings may also reflect inexperience with advocacy.

Consistent with these results, difficulties in understanding cost-sharing terminology such as deductible, coinsurance, annual benefit limit, and out-of-pocket maximum were reported by others [1]. A deductible is a fixed amount that must be paid by the insured each year before the insurance plan covers any additional costs [32]. A co-pay (co-payment) is an out-of-pocket fixed dollar amount or percentage that the insured pays for any medical service [32]. Coinsurance is a fixed percentage that the insured pays after meeting the deductible amount [32]. The out-of-pocket maximum is the total amount that an individual pays in a year toward covered medical expenses, after which the insurance provider will cover 100% of eligible services [32]. In the US, co-pays, deductibles, and coinsurance count toward the out-of-pocket maximum [32].

All respondents had stable health insurance coverage. These results may reflect the selection bias of the surveyed population comprising mainly NNPDF members. It may also imply that those respondents with access to NNPDF resources probably have job and financial security. Patients with childhood onset NPC were more likely to subscribe to a combination of Medicare/Medicaid/other public programs and private insurance and avail of a Medicaid waiver, whereas patients with ASMD were more likely to use private insurance only. Patients with NPC have significant and chronic neurological involvement that necessitates assistance with ADLs and require home-based care. This demand is anticipated to rise with an increase in disease awareness, improvements in diagnosis, and emergence of innovative disease-modifying interventions that can extend the life-span. Given the increased demand for symptomatic treatment, equipment (e.g., ambulatory aids, respiratory support devices), and services (e.g., caregiver assistance, physiotherapy, home schooling) in this group of patients, it is likely that caregivers would explore several options and avail of resources that provide maximum benefits to their children. Barring patients with infantile neurovisceral ASMD, most patients with ASMD do not have a significant neurological component and do not anticipate additional medical services; therefore, private insurance may be adequate to cover their medical expenses. Patients with adult onset NPC were most likely to use state-provided insurance and Medicaid waivers, indicating that they had possibly transitioned their health care to the state and were in the system long enough to avail of covered services and programs.

Most respondents (90%) did not have difficulty paying for their medical expenses; however, this may reflect lack of approved therapy or bias in the group that was studied. Often, respondents mentioned paying high co-pays for drugs, especially miglustat, which is used off-label and costs approximately $58,000 per month and may not be covered by waivers [33]. Yet, most respondents did not seek aid or subsidies from any reduced payment or financial assistance programs.

Follow-up interviews demonstrated that, regardless of NPD subtype, respondents faced substantial challenges to health care access. In general, all respondents perceived the process to obtain medical care and services as their greatest access challenge. Respondents expressed grave concerns about what the future would hold for them after the end of clinical trials that were providing medication and clinical expertise. These burdens included fear of access being delayed or denied, drug unavailability, prohibitive costs, and lack of access to medical experts in the field. Furthermore, respondents were uncertain about access to therapies such as miglustat that were approved in other countries but not in the US.

This study shows that despite high health insurance literacy among the NPD community, knowledge gaps exist. There remains an urgent need for guidance in navigating a complex health insurance system—a teaching opportunity for various organizations including patient-support organizations and advocacy groups. This could include providing easily digested educational aids (decision guides, programs for calculating out-of-pocket deductibles and maximums, financial tool kits, and consultancy services) and resources to enable individuals to assimilate health insurance terminology and concepts, compare and select from different plans, and better understand and utilize their existing plans to meet their unique needs and preferences. Educational decision aids that consider individual needs, provide cost estimates, and assess priorities were shown to improve individuals’ health insurance literacy and translated into self-confidence and more effective health insurance purchases [34]. These approaches are not limited to NPD but can be a collaborative effort that benefits other rare disease communities.

The study also illustrates the uncertainty among patients and caregivers regarding the accessibility, affordability, and availability of investigational drugs and medical services, especially after drug approval or when a clinical trial has ended. Access is especially challenging if the drug is not approved, in which case expanded access programs may be the only recourse for patients. Government-supported institutions, advocacy groups, and patient communities provide several resources and patient assistance programs to access essential medication and render health care services affordable; this information should be made available to the NPD community [35, 36]. Caregivers face immense pressure as they educate themselves and become medical experts, manage patient care across a plethora of specialties, connect with social service agencies for required services, and negotiate with insurance agencies for benefits. Caregivers constantly face the grim prospect of outliving the affected patient and have concerns on who will provide financial assistance and full-time care for the patient in their absence. Patient advocacy organizations can support caregivers by providing counseling services, connecting them with other patient families, and facilitating advocacy by strengthening and supporting their treatment and care decisions. These initiatives can ensure that patients have access to the cost savings, medical benefits, and health care services that they would otherwise not have access to or be aware of, especially in a postapproval era. Ultimately, the aim is to better serve the medical needs of patients with rare diseases, including NPD, and improve treatment outcomes.

### Study limitations

This study is subject to the limitations associated with surveys including recall bias or sharing inaccurate information. Members of the NPD community who engage with NNPDF are more likely to have participated in the survey. Therefore, unintentional selection biases may be introduced because of the individuals to whom the survey was sent and, consequently, because of those who responded to the survey. There is an inherent assumption that those who responded are representative of all members of NNPDF and, further, representative of members of the NPD community. These limitations may cause the results of the survey to be skewed, but the direction of skewing is unclear.

Furthermore, as patients with NPD were often children, respondents were caregivers, who can only provide proxy responses that may not accurately capture the patient’s voice.

## Conclusions

This study demonstrated a health insurance literacy gap and perceived access challenges among patients with NPD and their families. Lack of health insurance literacy and barriers to access medication and health care services can adversely impact health care utilization and outcomes. There is a critical unmet need to equip and prepare patients and caregivers with adequate educational programs, tools, and resources to comprehend and navigate complex health insurance processes and paperwork. This will empower them to make informed and personalized decisions regarding their health insurance plans and enable efficient utilization of health care services. This will facilitate access to quality treatment and services in a timely manner and prevent costly delays and disease progression. This study is the first step in identifying the needs of the NPD community. Findings from this study will be important for informing future initiatives to empower patients and caregivers with knowledge and skills to navigate a complicated health care system, understand their rights to access medication and services, and avail of resources and benefits to support living with NPD.

## Methods

### Study design and patients

This qualitative prospective observational cohort PRO study was conducted in the US by the NNPDF in collaboration with Engage Health from June 15, 2020, to September 21, 2020. Patients in the US NPD community (including members of NNPDF and other Niemann–Pick-related organizations, Engage Health’s EnCompass® database, and outreach to physicians treating patients with NPD) were invited via a written appeal to participate in this study. Recruitment material included social media posts and email outreach letters from patient associations and Engage Health. Study conduct was approved by the Western Institutional Review Board and Copernicus Group Institutional Review Board.

Patients with a confirmed diagnosis of any NPD subtype were eligible for study participation; NPD subtypes included Niemann–Pick type A/infantile neurovisceral ASMD, Niemann–Pick type A/B intermediate form or chronic neurovisceral ASMD, Niemann–Pick type B chronic visceral ASMD, Niemann–Pick type C early infantile form with an onset < 2 years of age, Niemann–Pick type C late infantile neurodegenerative form with onset at 2 to 6 years of age, Niemann–Pick type C juvenile neurodegenerative form with onset at 6 to 15 years of age, and Niemann–Pick type C adult neurodegenerative form with onset at > 15 years of age. Diagnosis of NPD was validated by NNPDF membership or proof of disease form. Patients aged ≥ 18 years or parent/legal guardian/caregiver of adult or minor patients with confirmed NPD who were able to read, write, and communicate in English and those who provided informed consent were included in the study. Parents with a child who had died within 2 years prior to the study (2018 or later) were also eligible for study participation.

### Survey

This 2-part study comprised an online quantitative survey to garner demographic and health insurance information and measure health insurance literacy as well as a qualitative, structured 30-min telephone interview on respondents’ perceptions of challenges in accessing health services. Only one respondent per family was permitted to participate in the study.

The health insurance survey was based on a 4-domain (knowledge, information-seeking, document literacy, and cognitive skills) health insurance literacy self-assessment tool that assessed an individual’s ability to purchase health insurance and utilize it [37]. The knowledge domain was assessed by a health insurance terminology and concepts quiz previously developed and used in a study by the Kaiser Family Foundation [38]. For the other domains, respondents rated themselves on their information-seeking skills (4 questions regarding, for example, knowing the location of information and navigation of insurance sources), document literacy skills (5 questions on, for example, filling out forms and understanding appeal processes), and cognitive skills (6 questions regarding, for example, applying how insurance relates to personal situations and utilization of services offered by insurance).

Patients or parents/caregivers, including those whose children had died within 2 years prior to the study, participated in a telephone interview. Parents with multiple children with NPD completed a separate interview for each child. Respondents were asked to share their views on the challenges they faced regarding access to and coverage of care and services, treatments and medications, and devices and equipment. Interviews were conducted using an aided followed by an unaided approach. Respondents also shared how these challenges impacted their daily living.

### Outcomes

The primary outcome measures of the study were health insurance literacy and health insurance coverage. Health insurance literacy was measured by the score received on a health insurance terminology quiz and self-ratings on respondents’ ability to navigate and understand insurance. Health insurance coverage was measured by multiple-choice and open-ended questions to capture insurance types and details of insurance plans such as co-pay and services covered.

### Statistical analysis

The health insurance literacy quiz was scored for correct responses out of a total of 10 questions; the number and percentage of correct responses for each question were recorded.

Data were analyzed using appropriate descriptive statistics such as frequency distributions, cross-tabulations, and measures of central tendency and dispersion. Continuous variables were analyzed for statistical significance using Pearson correlation. If statistically significant, the relationship between the variables was further analyzed using ordinary least squares regression. Categorical variables were defined using descriptive statistics. Cross-tabulations and *t* tests were used to test for statistical significance.

Anticipated access burdens were identified based on a review of the medical literature and defined. Respondents’ answers in the qualitative interview were weighted based on order and frequency of mention by the respondent. Weighted responses were categorized by 2 independent coders under the relevant access burden.

## Data Availability

The data set generated and/or analyzed over the course of this study is not publicly available but can be obtained from the corresponding author on request.

## Abbreviations

ADLs: Activities of daily living
ASMD: Acid sphingomyelinase deficiency
FDA: Food and Drug Administration
KFF: Kaiser Family Foundation
NNPDF: National Niemann–Pick Disease Foundation
NPA: Niemann–Pick disease type A
NPB: Niemann–Pick disease type B
NPC: Niemann–Pick disease type C
NPD: Niemann–Pick disease
OT: Occupational therapist
PROs: Patient-reported outcomes

## References

[1] Quincy L (2012). Measuring health insurance literacy: a call to action.

[2] Park S, Langellier BA, Meyers DJ (2022). Association of health insurance literacy with enrollment in traditional medicare, medicare advantage, and plan characteristics within medicare advantage. JAMA Netw Open.

[3] Ghaddar S (2021). Medicare for all: a health insurance literacy perspective. Health Lit Res Pract.

[4] Yagi BF, Luster JE, Scherer AM, Farron MR, Smith JE, Tipirneni R (2022). Association of health insurance literacy with health care utilization: a systematic review. J Gen Intern Med.

[5] Holland L, Nelson ML, Westrich K, Campbell PJ, Pickering MK (2021). The patient's medication access journey: a conceptual framework focused beyond adherence. J Manag Care Spec Pharm.

[6] Scotten M (2015). Parental health literacy and its impact on patient care. Prim Care.

[7] Levy H, Janke A (2016). Health literacy and access to care. J Health Commun.

[8] Braun RT, Barnes AJ, Hanoch Y, Federman AD (2018). Health literacy and plan choice: implications for medicare managed care. Health Lit Res Pract.

[9] Pasquini TLS, Goff SL, Whitehill JM (2021). Navigating the U.S. health insurance landscape for children with rare diseases: a qualitative study of parents' experiences. Orphanet J Rare Dis.

[10] Kaufmann P, Pariser AR, Austin C (2018). From scientific discovery to treatments for rare diseases—the view from the National Center for Advancing Translational Sciences—Office of Rare Diseases Research. Orphanet J Rare Dis.

[11] Tisdale A, Cutillo CM, Nathan R, Russo P, Laraway B, Haendel M (2021). The IDeaS initiative: pilot study to assess the impact of rare diseases on patients and healthcare systems. Orphanet J Rare Dis.

[12] Vanier MT (2010). Niemann–Pick disease type C. Orphanet J Rare Dis.

[13] Schuchman EH, Desnick RJ (2017). Types A and B Niemann–Pick disease. Mol Genet Metab.

[14] Wasserstein M, Dionisi-Vici C, Giugliani R, Hwu WL, Lidove O, Lukacs Z (2019). Recommendations for clinical monitoring of patients with acid sphingomyelinase deficiency (ASMD). Mol Genet Metab.

[15] Pinto C, Sousa D, Ghilas V, Dardis A, Scarpa M, Macedo MF (2021). Acid sphingomyelinase deficiency: a clinical and immunological perspective. Int J Mol Sci.

[16] McGovern MM, Dionisi-Vici C, Giugliani R, Hwu P, Lidove O, Lukacs Z (2017). Consensus recommendation for a diagnostic guideline for acid sphingomyelinase deficiency. Genet Med.

[17] McGovern MM, Aron A, Brodie SE, Desnick RJ, Wasserstein MP (2006). Natural history of Type A Niemann–Pick disease: possible endpoints for therapeutic trials. Neurology.

[18] Cassiman D, Packman S, Bembi B, Turkia HB, Al-Sayed M, Schiff M (2016). Cause of death in patients with chronic visceral and chronic neurovisceral acid sphingomyelinase deficiency (Niemann–Pick disease type B and B variant): literature review and report of new cases. Mol Genet Metab.

[19] McGovern MM, Lippa N, Bagiella E, Schuchman EH, Desnick RJ, Wasserstein MP (2013). Morbidity and mortality in type B Niemann–Pick disease. Genet Med.

[20] Berry-Kravis E (2021). Niemann–Pick disease, type C: diagnosis, management and disease-targeted therapies in development. Semin Pediatr Neurol.

[21] Mengel E, Patterson MC, Chladek M, Guldberg C (2021). Impacts and Burden of Niemann pick Type-C: a patient and caregiver perspective. Orphanet J Rare Dis.

[22] Geberhiwot T, Moro A, Dardis A, Ramaswami U, Sirrs S, Marfa MP (2018). Consensus clinical management guidelines for Niemann-Pick disease type C. Orphanet J Rare Dis.

[23] Cox GF, Clarke LA, Giugliani R, McGovern MM (2018). Burden of illness in acid sphingomyelinase deficiency: a retrospective chart review of 100 patients. JIMD Rep.

[24] Pokrzywinski R, Hareendran A, Nalysnyk L, Cowie S, Crowe J, Hopkin J (2021). Impact and burden of acid sphingomyelinase deficiency from a patient and caregiver perspective. Sci Rep.

[25] McGovern MM, Avetisyan R, Sanson BJ, Lidove O (2017). Disease manifestations and burden of illness in patients with acid sphingomyelinase deficiency (ASMD). Orphanet J Rare Dis.

[26] Imrie J, Galani C, Gairy K, Lock K, Hunsche E (2009). Cost of illness associated with Niemann–Pick disease type C in the UK. J Med Econ.

[27] Bajwa H, Azhar W. Niemann–Pick Disease. StatPearls. Treasure Island (FL);2022. 32310589

[28] Mayo Clinic. Niemann-Pick care at Mayo Clinic. https://www.mayoclinic.org/diseases-conditions/niemann-pick/care-at-mayo-clinic/mac-20355892. Accessed 13 July 2022.

[29] Sanofi. Press Release: Xenpozyme® (olipudase alfa) approved by European Commission as first and only treatment for ASMD 2022. https://www.sanofi.com/en/media-room/press-releases/2022/2022-06-28-05-30-00-2469974. Accessed 13 July 2022.

[30] Academy of Managed Care Pharmacy. Formulary Management 2019. https://www.amcp.org/about/managed-care-pharmacy-101/concepts-managed-care-pharmacy/formulary-management. Accessed 13 July 2022.

[31] Emily Johnson. What is a Medicaid waiver program? Medical News Today, 2021. https://www.medicalnewstoday.com/articles/medicaid-waiver-program#waiver-rules. Accessed 13 July 2022.

[32] Definitions of health insurance terms. https://www.bls.gov/ncs/ebs/sp/healthterms.pdf. Accessed 13 July 2022.

[33] Miglustat: Drug Information 2022. https://www.medilib.ir/uptodate/show/10244. Accessed 11 April 2022.

[34] Politi MC, Kuzemchak MD, Liu J, Barker AR, Peters E, Ubel PA, et al. Show me my health plans: using a decision aid to improve decisions in the federal health insurance marketplace. MDM Policy Pract. 2016;1. 10.1177/2381468316679998 PMC5550739 28804780

[35] Tips for finding financial aid: Genetic and Rare Diseases Information Center; updated May 28, 2020. https://rarediseases.info.nih.gov/Guides/pages/149/tips-for-finding-financial-aid. Accessed 11 Feb 2022.

[36] Financial Support: Every Life Foundation for Rare Diseases. https://everylifefoundation.org/financial-support/. Accessed 11 Feb 2022.

[37] Paez KA, Mallery CJ, Noel H, Pugliese C, McSorley VE, Lucado JL (2014). Development of the Health Insurance Literacy Measure (HILM): conceptualizing and measuring consumer ability to choose and use private health insurance. J Health Commun.

[38] Norton M, Hamel L. Assessing Americans' familiarity with health insurance terms and concepts: Kaiser Family Foundation; 2014. www.kff.org/health-reform/poll-finding/assessing-americans-familiarity-with-health-insurance-terms-and-concepts/. Accessed 10 Jan 2022.

